# Assessing the content and quality of gastrointestinal endoscopy videos on TikTok, Bilibili, and Kwai: a cross - sectional study

**DOI:** 10.1186/s12876-026-05108-6

**Published:** 2026-07-13

**Authors:** Jingsong Wang, Jing Wan, Bingxi Liu, Yixing Luo, Xu Shu, Linlin Liu, Zhenhua Zhu

**Affiliations:** 1 Department of Gastroenterology, Jiangxi Provincial Key Laboratory of Digestive Diseases, Jiangxi Clinical Research Center for Gastroenterology, Digestive Disease Hospital, The First Affiliated Hospital, Jiangxi Medical College, Nanchang University, Nanchang, Jiangxi China; 2 School of Medicine, Jianghan University, Wuhan, 430056 People’s Republic of China

**Keywords:** Gastrointestinal endoscopy, Video quality, Social media platforms, Health information, TikTok, Bilibili, Kwai

## Abstract

**Background:**

Gastrointestinal endoscopy is essential for diagnosing digestive diseases, yet public misconceptions persist. Short-video platforms (TikTok, Bilibili, Kwai) are now major health-information sources, but the quality of endoscopy-related content is inconsistent.

**Methods:**

We analyzed 300 endoscopy videos from the three platforms, categorizing uploaders as medical or non-medical professionals and scoring content with Global Quality Score (GQS), Journal of the American Medical Association (JAMA) benchmark criteria and Modified DISCERN scores across six dimensions (definition, eligibility, preparation, procedure, techniques, management).

**Results:**

Of the 300 videos analyzed, 63.3% were uploaded by medical professionals, with the highest proportion on TikTok (91.0%). Non-medical professionals contributed 36.7% of the videos, primarily on Bilibili and Kwai. TikTok and Kwai videos had higher user engagement (likes, comments, collections) but were shorter in duration compared to Bilibili videos. Quality assessments revealed significant differences among platforms, with TikTok videos scoring higher in reliability and accuracy (JAMA and Modified DISCERN scores) than Bilibili and Kwai videos. However, content completeness was generally low across all platforms, with most videos lacking comprehensive information on key aspects of gastrointestinal endoscopy.

**Conclusions:**

The quality and comprehensiveness of gastrointestinal endoscopy-related videos on TikTok, Bilibili, and Kwai are unsatisfactory, with significant variations in content quality and user engagement. Efforts should be intensified to improve video content quality, encourage more contributions from medical professionals, optimize algorithms to prioritize high-quality content, and implement stricter content reviews to enhance public health education and the dissemination of accurate medical information.

**Supplementary Information:**

The online version contains supplementary material available at https://doi.org/10.1186/s12876-026-05108-6.

## Introduction

With the rapid advancement of social media, short video sharing platforms have become pivotal mediums for disseminating information on various topics, including health-related content [1, 2]. In China, TikTok, Bilibili, and Kwai stand out as leading platforms, each with distinct user interfaces and functionalities. TikTok, known for its interactive, algorithm-driven short videos, appeals to younger audiences with content spanning entertainment, lifestyle, and education [3]. Bilibili, initially focused on animation and gaming, has evolved into a comprehensive platform targeting an intellectual audience, offering high-quality educational content, including health information, with an emphasis on credibility and evidence-based content [4]. Kwai, often seen as the grassroots counterpart to TikTok, attracts a broader audience, including rural and older demographics, with user-generated content that emphasizes personal stories [5]. However, the diversity of content on Kwai can lead to variations in the accuracy and professionalism of health-related videos [4, 6].

Among these platforms, gastrointestinal endoscopy has emerged as a topic of public interest. This medical examination method is crucial for observing the internal conditions of the digestive tract, from the esophagus to the duodenum [7, 8], and is essential for diagnosing and managing gastrointestinal diseases [9]. Endoscopy allows for direct observation and intervention, playing a key role in detecting diseases such as peptic ulcers, polyps, and malignant tumors [10, 11], and can perform simple therapeutic procedures like foreign body removal, hemostasis, and small tumor excision [12, 13]. Despite its clinical importance, many individuals lack understanding of endoscopy, leading to misconceptions, fear, or avoidance of the procedure [14–17].

In recent years, short videos related to gastrointestinal endoscopy have proliferated on these platforms [18, 19], presenting valuable opportunities for public education [20, 21]. However, the content quality varies significantly due to the range of video creators, from medical professionals to non-experts. Accurate and accessible health communication is essential to dispel misunderstandings, build public trust in medical procedures, and promote preventive practices [22–24]. While these platforms offer promising avenues for public health education, concerns about the reliability, accuracy, and comprehensiveness of information persist. Misleading or exaggerated content can lead to erroneous public behavior [1, 25], erode trust in medical practices, or spread inaccurate information [26, 27]. Despite the growing influence of these platforms, systematic assessments of short videos related to gastrointestinal endoscopy on these major Chinese platforms remain limited.

Therefore, this study aims to evaluate the comprehensiveness, accuracy, and scientific nature of information related to gastrointestinal endoscopy on TikTok, Bilibili, and Kwai. By identifying the strengths and weaknesses of these platforms, we aim to propose recommendations for improvement. Through this research, we hope to provide an empirical basis for enhancing health education content on these platforms, facilitate public access to high-quality medical information, and offer valuable insights for future policy-making and academic research.

## Methods

### Search strategy

**Fig. 1:**
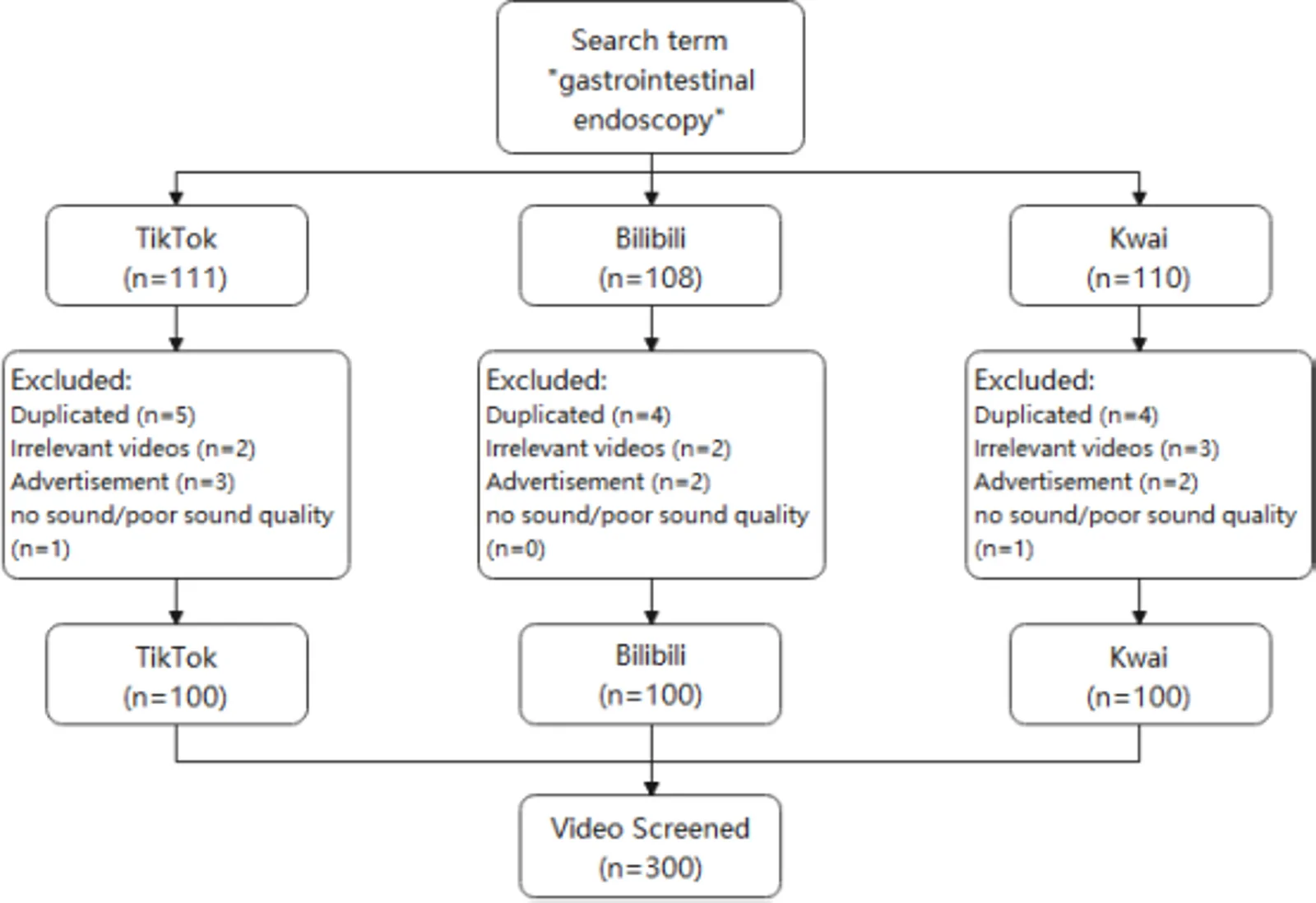
Flowchart of patients included in this study

To ensure the objectivity and impartiality of the study, we selected three major short video platforms in China: Bilibili, TikTok, and Kwai, as our data sources. On each platform, we used the keyword “gastrointestinal endoscopy” to search and selected the top 100 highest-rated relevant videos for analysis (Fig. 1). To avoid potential biases in search results caused by personalized algorithms, we logged out of all user accounts and cleared browsing history before conducting the search. The video collection was conducted on July 25, 2024. Considering that the number of views and likes of videos may fluctuate over time, we included only those uploaded before July 11, 2024. In the video selection process, we performed keyword searches according to the default order of the websites. Subsequently, two researchers independently assessed the content quality and information accuracy of the selected videos. In case of discrepancies in the assessment results, the researchers would reach a consensus through discussion or by consulting the corresponding author. The following were the inclusion and exclusion criteria: Inclusion criteria: (1) Videos directly related to gastrointestinal endoscopy procedures; (2) Videos in Chinese language; (3) Videos uploaded before July 11, 2024. Exclusion criteria: (1) Videos with duplicate content; (2) Videos primarily used for promotion and commercialization; (3) Videos with incomplete information records; (4) Videos with severe audio quality issues that affect information delivery. These measures were taken to ensure the relevance and high quality of the analyzed videos, thereby guaranteeing the scientific nature and practical value of the study results.

### Video characteristics

In this study, we meticulously extracted and documented various characteristics of the videos, including views, likes, comments, collections, shares, duration, upload date, source of upload, and the video content itself, alongside evaluations using the Global Quality Score (GQS), the Journal of the American Medical Association (JAMA) benchmark criteria, and the Modified DISCERN score. Based on the details provided in the uploader’s account profile, such as the account name and verification status, and the nature of content shown, videos were categorized into two principal groups: Medical professionals (including gastroenterologists and non-gastroenterologists) and Nonmedical professionals (including general users, news agencies/nonprofit organizations, and science communicators).

For assessing the video content, we relied on three extensively used standardized scales: GQS, JAMA, and Modified DISCERN. These instruments were instrumental in evaluating the quality and efficacy of the videos. The GQS is employed to gauge the quality of health information in videos, rating aspects such as clarity, depth, and utility to patients on a 5-point scale where 1 stands for poor quality and 5 for excellent quality and clarity (Supplementary Table 1) [28–30]. The JAMA score quantifies the reliability of videos based on four essential criteria: disclosure of authorship, citation of references and content sources including copyright information, clear indication of the posting and update dates, and the disclosure of any potential conflicts of interest such as funding or sponsorship (Supplementary Table 2) [31, 32]. Each criterion scores 1 point, leading to a maximum score of 4. The Modified DISCERN evaluates videos through a set of five questions assessing clarity, reliability of information sources, balance and fairness of information, inclusion of extra resources for patients, and the treatment of uncertainties or controversies in the content. Each affirmative answer yields 1 point, with the total also culminating in 5 points (Supplementary Table 3) [33].

Additionally, the breadth of video content was measured across six primary dimensions: definition, eligibility, preparation, procedure, techniques, and management. Based on how well the video content addressed these aspects, videos were rated in five tiers: no content (0 points), minimal content (0.5 points), moderate content (1 point), substantial content (1.5 points), and extensive content (2 points).

### Statistical analysis

For the statistical evaluation of data in this study, we utilized SPSS software, version 27.0 (IBM, Chicago, IL, USA) and the R statistical package, version 4.3.1. Quantitative data that followed a normal distribution were summarized using the mean and standard deviation (mean ± SD) and analyzed with the t-test to identify significant differences. For data that did not follow a normal distribution, results were described using the median and interquartile range (IQR), and the Mann-Whitney U test was used to assess differences between groups. Categorical data were presented as frequencies and percentages. The chi-square test was employed to determine differences among categorical variables, and Fisher’s exact test was used when appropriate, particularly for small sample sizes. A p-value of less than 0.05 was considered statistically significant.

## Results

**Fig. 2:**
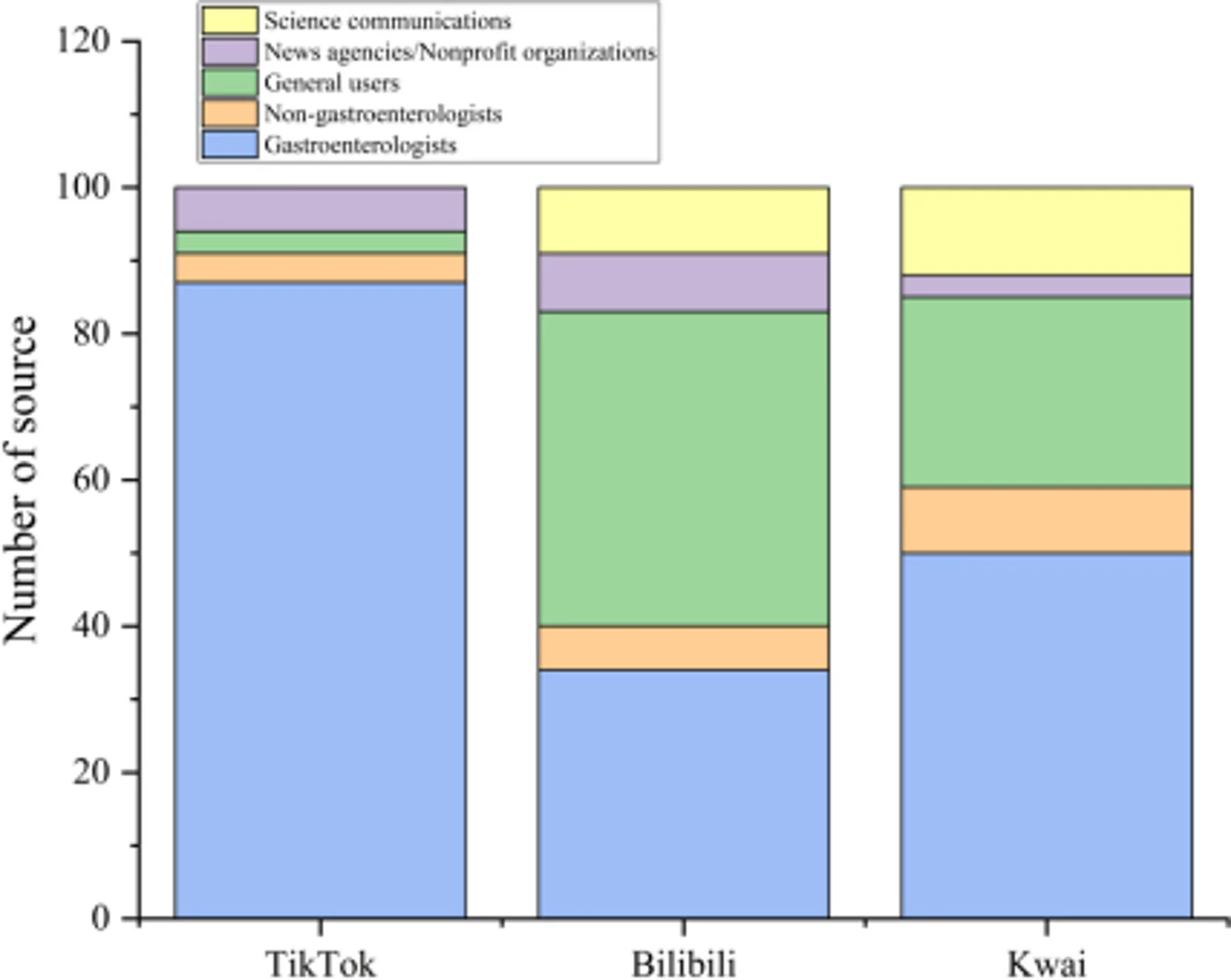
Video sources for the three platforms

The baseline characteristics of video content across different platforms (TikTok, Bilibili, and Kwai) were summarized and statistically analyzed (Table 1). The distribution of video sources varied significantly among the platforms (*P* = 0.001). Videos from medical professionals accounted for 63.3% of the total, with the highest proportion on TikTok (91.0%), followed by Kwai (59.0%) and Bilibili (40.0%) (Fig. 2). Among medical professionals, gastroenterologists were the primary contributors, comprising 57.0% of all videos, with 87.0% from TikTok, 50.0% from Kwai, and 34.0% from Bilibili. Non-medical professionals contributed 36.7% of the videos, mainly from Bilibili (60.0%) and Kwai (41.0%), while TikTok had only 9.0%. General users were responsible for 24.0% of the videos, predominantly on Bilibili (43.0%) and Kwai (26.0%), with minimal representation on TikTok (3.0%). Science communication videos were entirely absent from TikTok but accounted for 9.0% and 12.0% of videos on Bilibili and Kwai, respectively.

**Table 1:** Comparison of source and scores of videos about gastrointestinal endoscopy on three different platforms

Parameters	Total	TikTok	Bilibili	Kwai	*P*
Video source					<0.01
Medical professionals	190(63.3%)	91(91.0%)	40(40.0%)	59(59.0%)	
Gastroenterologists	171(57.0%)	87(87.0%)	34(34.0%)	50(50.0%)	
Non-gastroenterologists	19(6.3%)	4(4.0%)	6(6.0%)	9(9.0%)	
Nonmedical professionals	110(36.7%)	9(9.0%)	60(60.0%)	41(41.0%)	
General users	72(24.0%)	3(3.0%)	43(43.0%)	26(26.0%)	
News agencies/Nonprofit organizations	17(5.7%)	6(6.0%)	8(8.0%)	3(3.0%)	
Science communications	21(7.0%)	0	9(9.0%)	12(12.0%)	
Views	/	/	3714.0(1028.5-11500.0)	/	/
Number of likes	246.0(61.5–1237.0)	573.0(148.0-2230.5)	49.0(12.0-154.0)	482.5(117.5–2384.0)	0.099
Number of comments	33.5(6.0-219.5)	67.0(15.0-883.5)	5.5(1.0-25.5)	70.0(12.5–451.0)	0.027
Number of collections	84.5(22.0-282.0)	100.5(30.0-425.5)	50.0(19.0-200.0)	100.5(20.5–343.0)	0.045
Number of favorites	/	77.5(22.0-345.0)	18.5(6.0–62.0)	/	/
Duration (s)	99.5(42.0-279.5)	61.5(24.5-105.5)	549.0(230.5-1369.5)	62.0(33.5–112.0)	0.001
Time since upload (d)	476.0(265.5-816.5)	406.0(208.0-710.5)	495.5(271.5–1021.0)	490.0(291.5-789.5)	0.02
GQS	2.0(2.0–3.0)	2.0(2.0–3.0)	3.0(2.0–3.0)	3.0(2.0–3.0)	0.103
JAMA	2.0(0–3.0)	2.0(1.0–2.0)	1.0(0–3.0)	1.0(0–2.0)	0.001
Modified DISCERN	2.0(1.0–3.0)	2.0(2.0-3.5)	1.0(1.0–3.0)	2.0(1.0–4.0)	0.001

In terms of user engagement metrics, there was no significant difference in the number of likes among platforms (*P* = 0.099), but the median values varied significantly. TikTok videos had the highest median number of likes [573.0 (148.0–2230.5)], followed by Kwai [482.5 (117.5–2384.0)] and Bilibili [49.0 (12.0–154.0)], reflecting higher user engagement on short-video platforms like TikTok and Kwai. The number of comments differed significantly across platforms (*P* = 0.027), with Kwai having the highest median number of comments [70.0 (12.5–451.0)], closely followed by TikTok [67.0 (15.0–883.5)], while Bilibili had considerably fewer comments [5.5 (1.0–25.5)]. Similarly, the number of collections also varied significantly (*P* = 0.045), with both TikTok [100.5 (30.0–425.5)] and Kwai [100.5 (20.5–343.0)] outperforming Bilibili [50.0 (19.0–200.0)]. These results indicate that videos on TikTok and Kwai not only garnered more likes but also encouraged higher levels of active interaction through comments and collections compared to Bilibili.

**Fig. 3:**
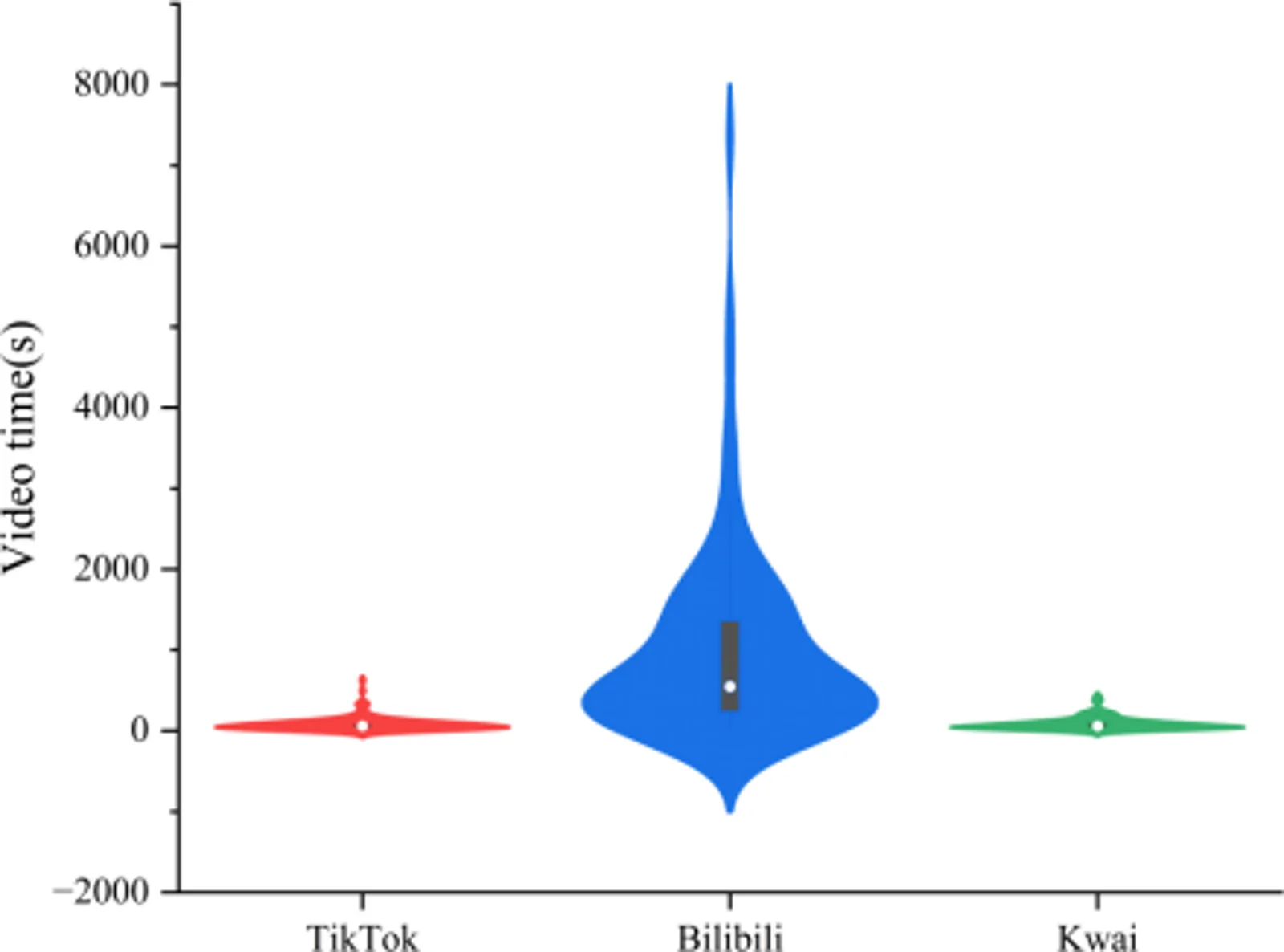
Video times for the three platforms

**Fig. 4:**
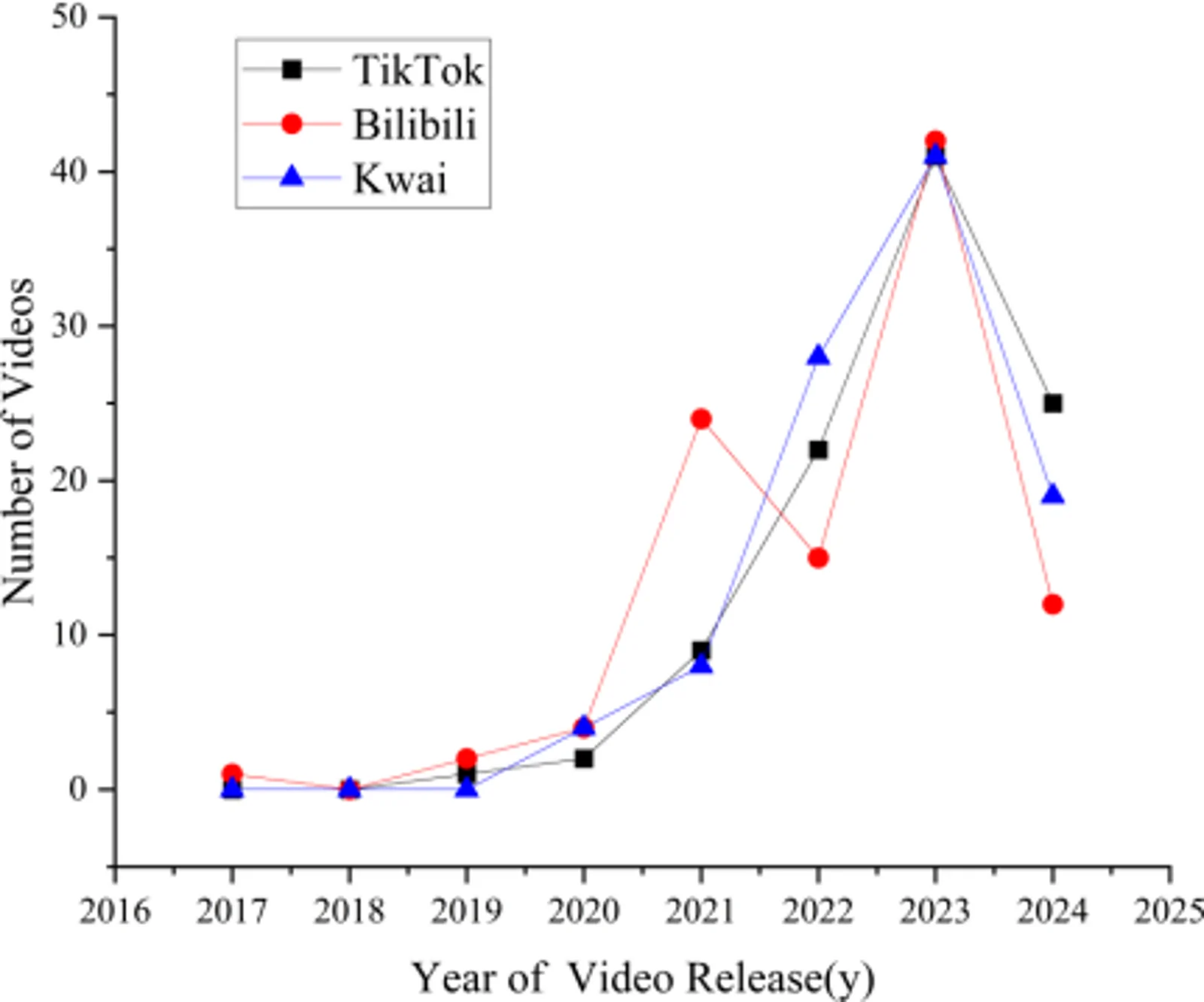
Gastrointestinal endoscopy-related videos published on various video platforms between 2017 and 2024

**Fig. 5:**
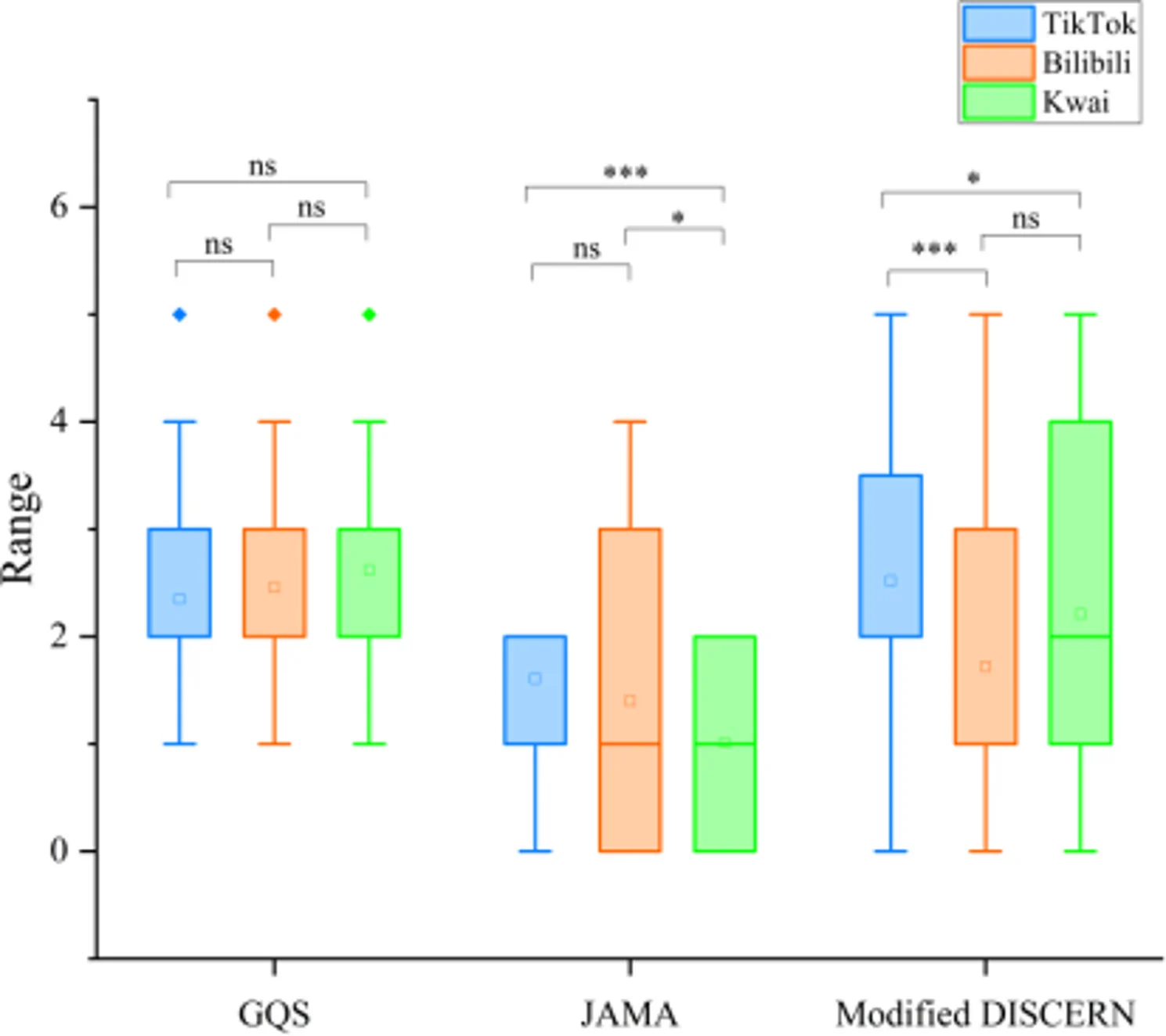
Comparison of GQS, JAMA, and Modified DISCERN scores on three video platforms

Video duration differed markedly among platforms (*P* = 0.001), with Bilibili videos being significantly longer [549.0 s (230.5–1369.5)] compared to Kwai [62.0 s (33.5–112.0)] and TikTok [61.5 s (24.5–105.5)] (Fig. 3). The time since upload also varied significantly (*P* = 0.02), with Bilibili videos being the oldest [495.5 days (271.5–1021.0)], followed by Kwai [490.0 days (291.5–789.5)] and TikTok [406.0 days (208.0–710.5)]. Figure 4 presents the number of videos released each year on the three platforms. There was a noticeable increase in the number of videos from 2020 to 2023, with a peak in 2023, after which the number of videos declined. In terms of video quality assessment, the Global Quality Score (GQS) showed no significant differences among platforms (*P* = 0.103), with a median score of 2.0 (2.0–3.0) across all videos (Fig. 5). However, significant differences were observed in JAMA and Modified DISCERN scores (*P* = 0.001 for both). TikTok videos had the highest JAMA scores [2.0 (1.0–2.0)], outperforming videos from Bilibili and Kwai, both of which had median scores of 1.0 (ranges 0–3.0 and 0–2.0, respectively). Similarly, TikTok videos achieved higher Modified DISCERN scores [2.0 (2.0–3.5)], comparable to Kwai [2.0 (1.0–4.0)] and superior to Bilibili [1.0 (1.0–3.0)], suggesting that videos on TikTok and Kwai generally offered more reliable and higher-quality information compared to those on Bilibili.

**Fig. 6:**
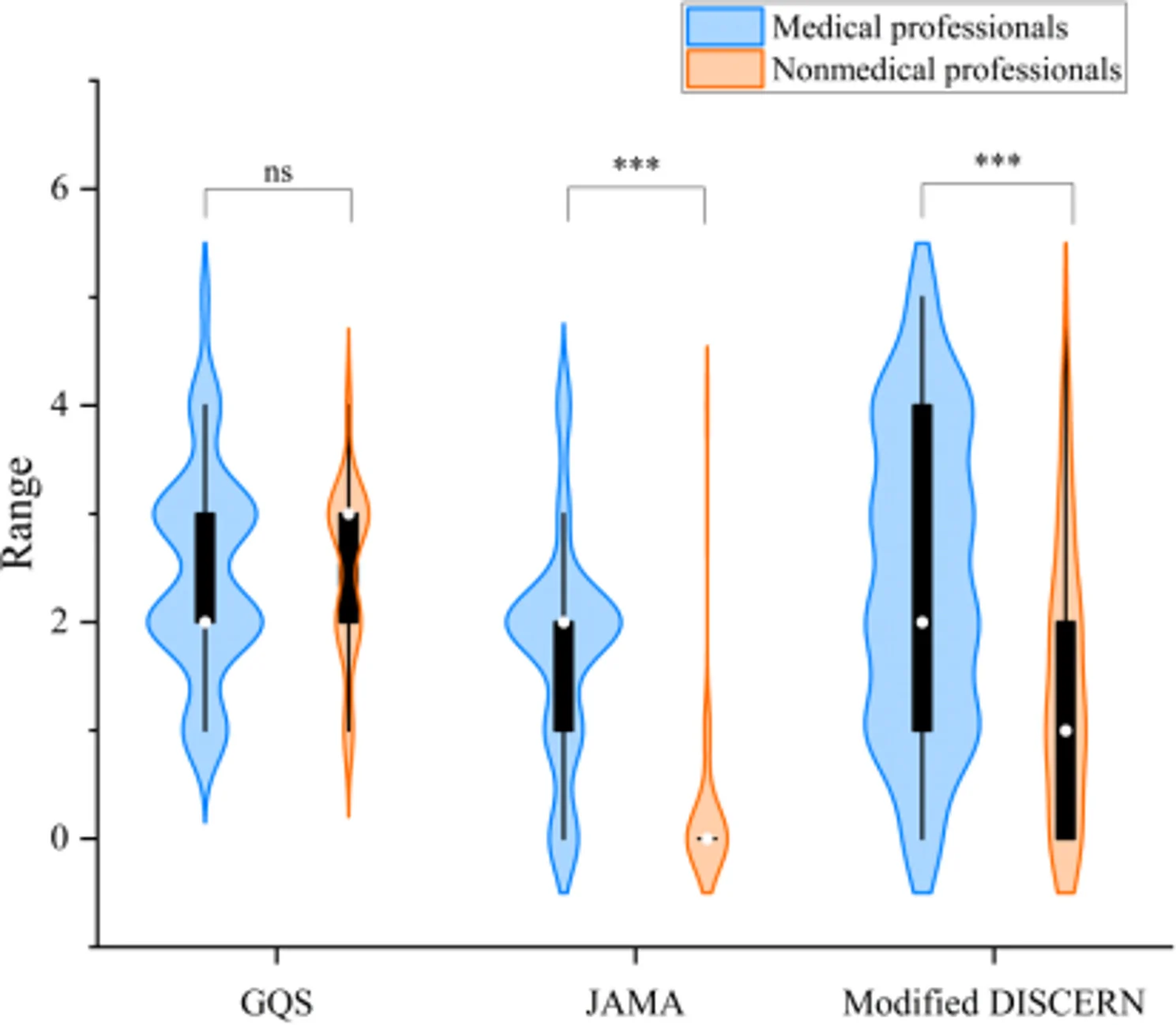
Comparison of GQS, JAMA, and Modified DISCERN scores on different video source

**Fig. 7:**
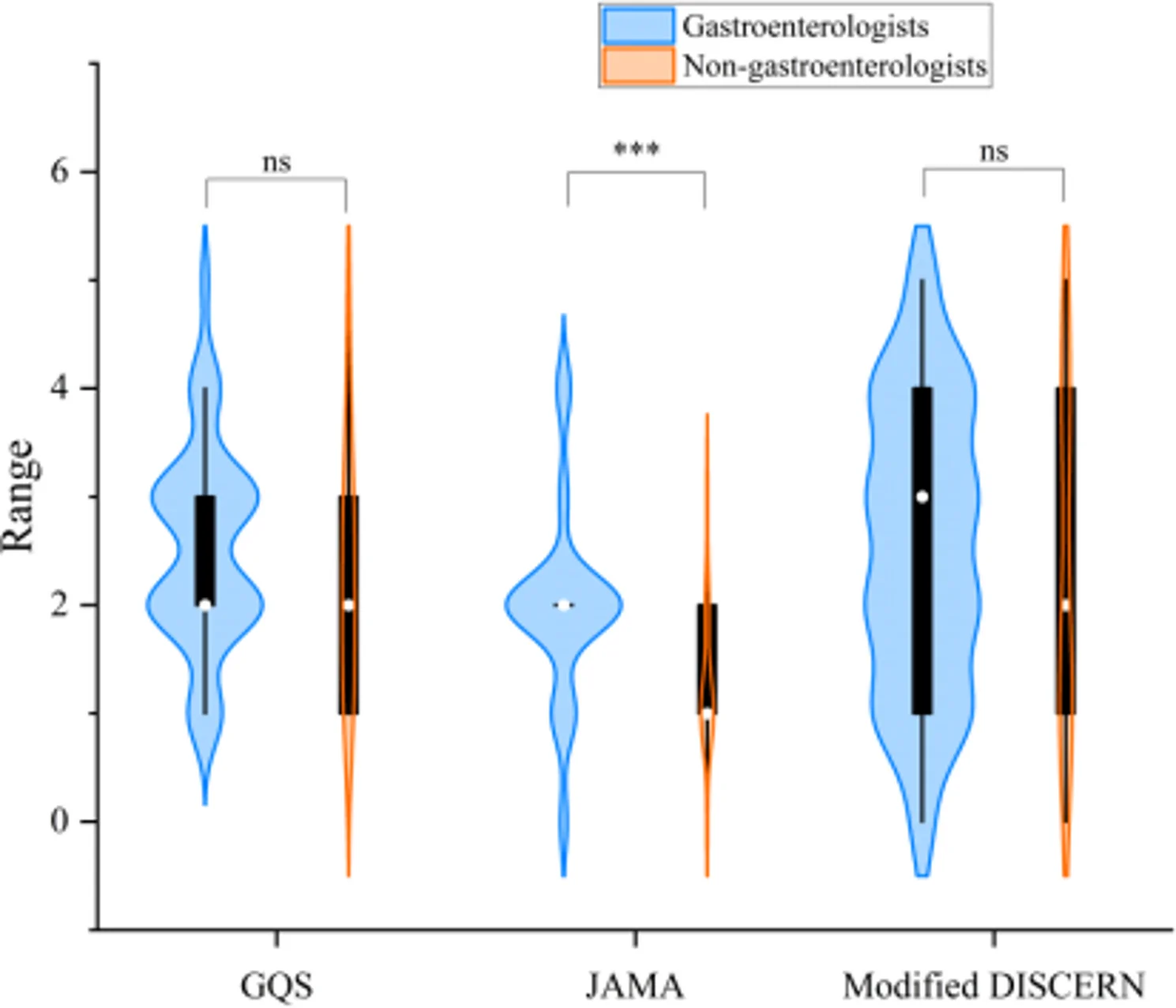
Comparison of GQS, JAMA, and Modified DISCERN scores between gastroenterologists and non-gastroenterologists

Further analysis of the video features based on different sources revealed significant differences in several aspects (Table 2), with p-values indicating statistical significance (*p* < 0.05). Videos created by medical professionals, especially gastroenterologists, generally had higher numbers of likes, comments, and collections compared to those created by non-medical professionals (Figs. 6 and 7). For example, gastroenterologists’ videos had a median number of likes of 243.0 (83.5–1358.5), while general users’ videos had 339.5 (55.0–1581.0). The duration of videos also varied significantly among different sources, with general users’ videos being the longest [237.5 s (129.0–490.5)] and non-gastroenterologists’ videos being the shortest [57.0 s (26.5–224.0)]. In terms of quality assessment, videos from medical professionals had higher GQS, JAMA, and Modified DISCERN scores, indicating better quality and reliability. For instance, gastroenterologists’ videos had a median JAMA score of 2.0 (2.0–2.0), while non-medical professionals’ videos had lower scores, with general users’ videos having a median JAMA score of 0. The differences in these aspects were statistically significant (*p* < 0.05). Additionally, videos from news agencies/nonprofit organizations and science communications had varying levels of engagement and quality, but overall, medical professionals’ videos outperformed non-medical professionals’ videos in terms of user interaction and content quality.

**Table 2:** Comparison of video features on different video source

Video source	Medical professionals		Nonmedical professionals			*P*
	Gastroenterologists	Non-gastroenterologists	General users	News agencies/Nonprofit organizations	Science communications	
Number of likes	243.0(83.5-1358.5)	201.0(52.0-830.0)	339.5(55.0-1581.0)	164.0(40.0-1411.0)	71.0(23.0-442.0)	0.718
Number of comments	30.0(6.0-239.0)	21.0(6.0-214.0)	58.0(10.0-385.5)	11.0(1.0-117.0)	10.0(3.0–77.0)	0.728
Number of collections	76.0(22.0-260.5)	103.0(19.0-387.0)	99.5(26.5–400.0)	131.0(24.0-425.0)	57.0(19.0-230.0)	0.392
Duration (s)	76.0(36.0-152.0)	57.0(26.5–224.0)	237.5(129.0-490.5)	105.0(38.0-144.0)	75.0(36.0-216.0)	0.001
Time since upload (d)	469.0(273.5-803.5)	406.0(222.0-536.5)	518.5(200.0-938.0)	453.0(292.0-880.0)	504.0(314.0-795.0)	0.338
GQS	2.0(2.0–3.0)	2.0(1.5-3.0)	2.5(2.0–3.0)	3.0(2.0–3.0)	2.0(2.0–3.0)	0.667
JAMA	2.0(2.0–2.0)	1.0(1.0-1.5)	0	0(0–1.0)	0(0–2.0)	0.001
Modified DISCERN	3.0(1.0–4.0)	2.0(1.0–4.0)	1.0(0–2.0)	2.0(1.0–3.0)	1.0(1.0–3.0)	0.001

**Fig. 8:**
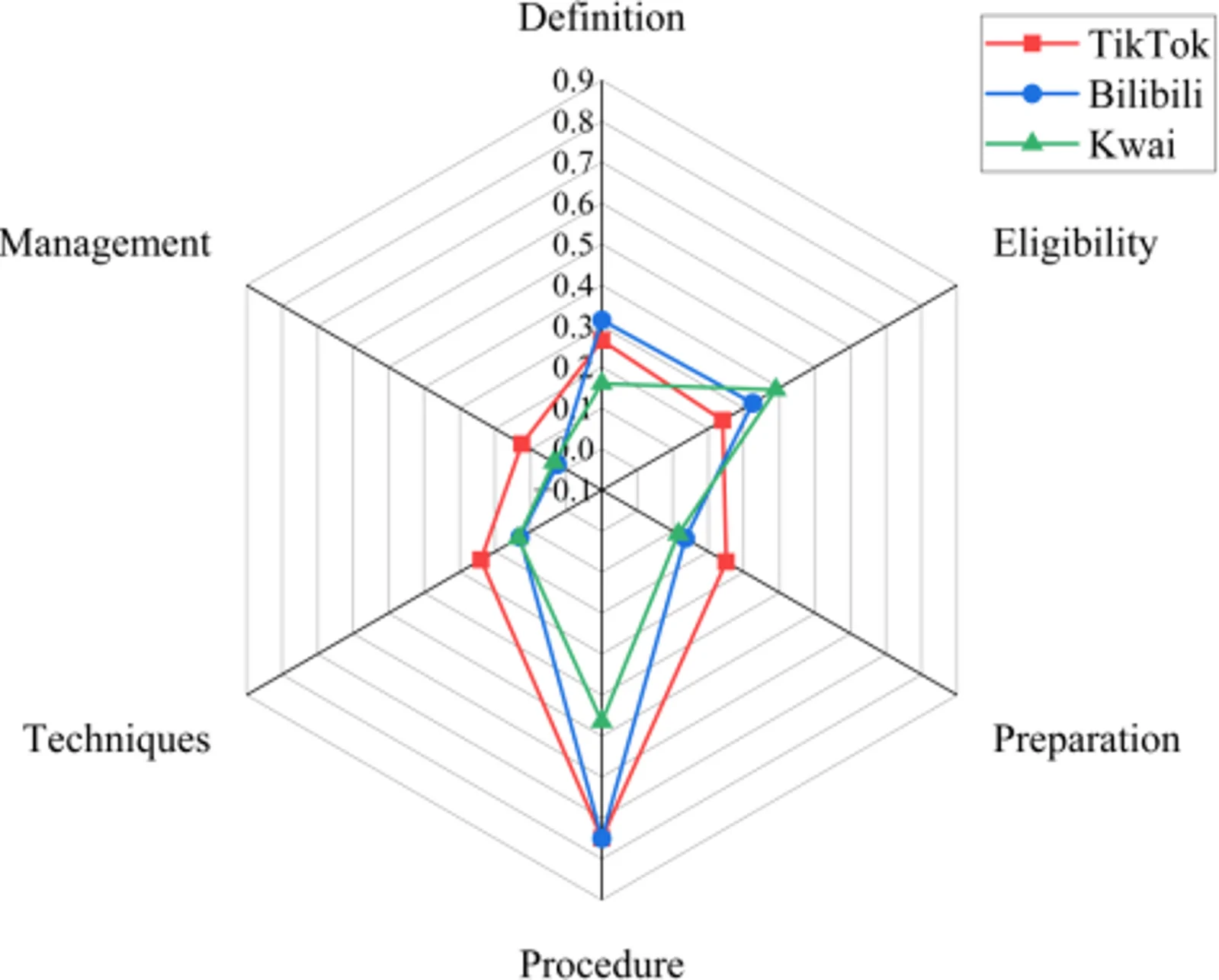
Comparison of content comprehensiveness on three video platforms

The completeness of video content was assessed across six dimensions: definition, eligibility, preparation, procedure, techniques, and management (Table 3; Fig. 8). The results showed that the overall completeness of content was low across all platforms. For example, on TikTok, 81% of videos had no content on the definition of gastrointestinal endoscopy, and 87% had no content on outcomes. Similar trends were observed on Bilibili and Kwai, indicating a significant gap in the comprehensiveness of information provided.

**Table 3:** Completeness of video content

	Video content	Definition	Eligibility	Preparation	Techniques	Management	Outcome
TikTok	No content (0 points)	81(81%)	73(73%)	70(70%)	36(36%)	77(77%)	87(87%)
	Little content (0.5 points)	3(3%)	12(12%)	18(18%)	12(12%)	9(9%)	6(6%)
	Some content (1 point)	4(4%)	10(10%)	7(7%)	30(30%)	8(8%)	4(4%)
	Most content (1.5 points)	6(6%)	4(4%)	2(2%)	10(10%)	1(1%)	1(1%)
	Extensive content (2 points)	6(6%)	1(1%)	3(3%)	12(12%)	5(5%)	2(2%)
Bilibili	No content (0 points)	71(71%)	58(58%)	81(81%)	24(24%)	83(83%)	95(95%)
	Little content (0.5 points)	8(8%)	21(21%)	12(12%)	29(29%)	10(10%)	5(5%)
	Some content (1 point)	13(13%)	19(19%)	6(6%)	27(27%)	5(5%)	0
	Most content (1.5 points)	3(3%)	2(2%)	1(1%)	13(13%)	2(2%)	0
	Extensive content (2 points)	5(5%)	0	0	7(7%)	0	0
Kwai	No content (0 points)	83(83%)	44(44%)	84(84%)	31(31%)	82(82%)	95(95%)
	Little content (0.5 points)	8(8%)	34(34%)	11(11%)	47(47%)	9(9%)	3(3%)
	Some content (1 point)	5(5%)	22(22%)	4(4%)	20(20%)	9(9%)	2(2%)
	Most content (1.5 points)	2(2%)	0	0	2(2%)	0	0
	Extensive content (2 points)	2(2%)	0	1(1%)	0	0	0

The correlation analysis of the statistical data revealed significant positive correlations between the Modified DISCERN scores and both the GQS scores (*r* = 0.646, *p* = 0.001) and the JAMA scores (*r* = 0.288, *p* = 0.001) (Table 4). The GQS scores were positively associated with video duration (*r* = 0.196, *p* = 0.001), while the Modified DISCERN scores were inversely related to video duration (*r* = -0.134, *p* = 0.02). Additionally, the JAMA scores were negatively correlated with the number of comments (*r* = -0.168, *p* = 0.004). No statistically significant correlations were found between any of the quality assessment scores (JAMA, GQS, Modified DISCERN) and the metrics of likes or collections.

**Table 4:** Correlation analysis

	Likes	Comments	Collections	Duration	Time since upload	GQS	JAMA	Modified DISCERN
GQS								
r	0.094	0.075	0.094	0.196	0.018	-	0.056	0.646
p value	0.105	0.194	0.103	0.001	0.757	-	0.334	0.001
JAMA								
r	-0.1	-0.168	0.011	-0.003	-0.082	0.056	-	0.288
p value	0.083	0.004	0.850	0.955	0.154	0.334	-	0.001
Modified DISCERN								
r	0.087	0.064	-0.037	-0.134	0.034	0.646	0.288	-
p value	0.131	0.268	0.523	0.020	0.556	0.001	0.001	-

## Discussion

This study evaluated 300 gastrointestinal endoscopy-related videos across TikTok, Bilibili, and Kwai, revealing several key findings. First, medical professionals uploaded 63.3% of the videos, with TikTok having the highest proportion of medically authored content (91.0%), while Bilibili was dominated by non-medical creators (60.0%). Second, TikTok and Kwai videos demonstrated higher user engagement (likes, comments, and collections) but were significantly shorter in duration compared to Bilibili videos. Third, quality assessments showed that TikTok videos achieved higher reliability scores (median JAMA score: 2.0; median Modified DISCERN score: 2.0) than Bilibili (median JAMA: 1.0; median Modified DISCERN: 1.0) and Kwai (median JAMA: 1.0; median Modified DISCERN: 2.0) videos. Fourth, content completeness was generally poor across all platforms, with over 80% of videos lacking comprehensive information on key aspects such as procedure definition, eligibility criteria, preparation requirements, and post-procedure management. These findings underscore the need for improved health education content on social media platforms [5, 25].

Gastrointestinal endoscopy is the cornerstone for diagnosing and treating a variety of digestive system diseases, such as peptic ulcers, polyps, and malignant tumors. This minimally invasive examination method can directly observe the inside of the gastrointestinal tract and perform therapeutic interventions, significantly improving the early detection rate and treatment outcomes of diseases. Despite its clinical indispensability, public awareness and understanding of gastrointestinal endoscopy remain insufficient, leading to widespread misunderstandings, fear, and avoidance of the procedure [14]. These knowledge gaps hinder timely medical consultation and adherence to recommended screenings, especially in high-risk populations. The increasing prevalence of gastrointestinal diseases, influenced by factors such as population aging, changes in dietary structure, and environmental alterations, underscores the urgency of improving public health literacy regarding gastrointestinal endoscopy. However, in China, strained medical resources and limited personalized patient education pathways have intensified people’s reliance on digital platforms for medical information [3].

The rapid development of short video platforms such as TikTok, Bilibili, and Kwai has completely changed the way medical information is disseminated, offering unprecedented convenience for the public to access health-related content. However, our analysis of gastrointestinal endoscopy-related videos indicates significant differences in content quality and user engagement patterns across these platforms, which highlights both their potential as tools for public health education and their limitations.

Our analysis shows that videos from health professionals, especially gastroenterologists, performed better in JAMA and Modified DISCERN scores, reflecting their higher reliability and scientific accuracy. However, such videos only account for a small proportion of the total content (for example, videos made by gastroenterologists account for 57% on TikTok, but only 34% on Bilibili). Videos made by non-medical contributors, including general users and science bloggers, account for nearly half of the analyzed videos, many of which oversimplify explanations, exaggerate procedural risks, or provide incomplete coverage of key topics such as pre- and post-procedure care. Specifically, we identified several categories of misleading claims in non-professional videos: (1) exaggerated descriptions of procedural pain and discomfort, which may deter patients from undergoing necessary diagnostic or therapeutic endoscopies; (2) oversimplified or inaccurate explanations of procedural risks, potentially causing either unnecessary anxiety or false reassurance; (3) promotion of unproven alternative diagnostic methods or dietary supplements as substitutes for endoscopic examination; and (4) incomplete or absent information on critical pre-procedure preparation requirements (e.g., fasting protocols, medication adjustments), which could lead to procedure cancellations or complications. These misleading claims pose multifaceted harms: for patients, they may engender fear, delay seeking appropriate care, or foster mistrust in medical recommendations; for healthcare providers, they create additional burden in correcting misconceptions during clinical consultations; for hospitals and healthcare systems, they may contribute to lower screening adherence rates and delayed diagnoses, ultimately affecting population health outcomes.

Wang et al. [34] evaluated GI bleeding videos on Bilibili, TikTok, and YouTube using identical instruments (GQS, JAMA, Modified DISCERN) and a comparable sample (*n* = 300). Their findings closely align with ours: approximately 65% of videos were uploaded by health professionals, TikTok had the highest proportion of medical content, and overall quality scores were similarly low (median DISCERN: 2; GQS: 3). Both studies found that professional videos outperformed non-professional uploads in reliability. A notable divergence lies in content comprehensiveness: Wang et al. reported no significant difference between professional and non-professional videos [34], whereas our study showed that medical professionals’ videos covered more procedural aspects. This may reflect the differing nature of the topics—GI bleeding is a broad clinical manifestation, whereas endoscopy is a specific intervention with standardized preparation and management protocols.

Similarly, Wang et al. [35] assessed gastric cancer videos on the same three platforms using comparable instruments. They reported suboptimal information quality with significant cross-platform variation, consistent with our findings. Videos from verified medical professionals achieved higher JAMA and DISCERN scores, while user-generated content frequently contained incomplete or misleading information. Wang et al. also observed that longer Bilibili videos did not guarantee better quality due to non-professional dominance [35], a pattern we similarly identified. The concordance across these studies suggests that the challenges facing health information quality on Chinese short-video platforms are not topic-specific but represent a systemic issue spanning multiple disease domains.

Platform-specific differences further complicate the situation. TikTok and Kwai, which focus on short video formats (with a median duration of about 60 s), showed higher user engagement metrics (likes, comments, and collections), but their content often emphasized brevity over depth. For example, over 80% of TikTok videos lacked key information on symptoms, risk factors, and treatment outcomes—a trend also seen on Kwai and Bilibili. Although Bilibili’s videos were longer (with a median duration of about 549 s), theoretically allowing for more detailed explanations, their quality did not significantly outperform short videos due to the dominance of non-professional creators (60% of Bilibili’s videos were made by non-professionals). This finding reflects the differences in content format and user behavior preferences across platforms, as Bilibili tends to feature long-form content while TikTok and Kwai emphasize shorter, more concise videos. This contradiction highlights a key challenge: even with longer video duration, comprehensive content cannot be guaranteed without professional involvement.

The widespread presence of incomplete or misleading information poses real risks. For example, some non-gastroenterologist creators exaggerated the discomfort of endoscopy, which might deter patients from necessary screenings. In contrast, videos made by gastroenterologists, though more accurate, often focused on lifestyle changes (such as dietary adjustments) while omitting crucial details on procedural aspects, testing plans, and long-term prognosis that are vital for patients to make informed consent and cooperate with treatment. This imbalance reflects broader systemic issues, such as algorithms prioritizing interactivity over accuracy and resource limitations preventing healthcare professionals from producing in-depth content [12].

Short videos can make complex health information easier to understand and more memorable. Therefore, improving the quality of information in short videos is crucial for maintaining patient health and reducing the economic burden on patients. To this end, we propose the following suggestions to make short videos a truly important tool for future health education. First, platforms should incentivize medical professionals, especially gastroenterologists, to create and share high-quality content. This could include partnerships with healthcare organizations, financial incentives, or training programs to help professionals produce engaging and informative videos. Second, platforms should adjust their recommendation algorithms to prioritize scientifically accurate and comprehensive content over highly interactive but potentially misleading videos. This could be achieved by giving greater weight to videos from verified medical professionals and those covering key educational topics. Finally, stricter content review mechanisms should be implemented to ensure that health-related videos meet minimum standards of accuracy and reliability. This could involve collaborating with medical experts to verify the scientific validity of content before it is published.

### Limitations

This study has some limitations. First, it focused only on Chinese video content, which may limit the generalizability of the findings to other linguistic or cultural contexts. Second, the evaluation of video quality was limited to three major platforms, and the quality of videos on other platforms requires further investigation. Third, interobserver agreement statistics were not systematically captured during the independent scoring phase, which limits our ability to report the reliability of the quality assessment process. Lastly, due to platform restrictions, some metrics such as view counts and shares were not available, which could have provided additional insights into user engagement.

## Conclusion

This study evaluated gastrointestinal endoscopy-related videos on TikTok, Bilibili, and Kwai, revealing significant differences in content quality, engagement, and completeness. While TikTok and Kwai showed higher user engagement and better content reliability, Bilibili had lower engagement and quality despite longer videos. Overall, content completeness was poor across all platforms, with most videos lacking comprehensive information. To improve health education on these platforms, it is crucial to encourage more contributions from medical professionals, optimize algorithms to prioritize high-quality content, and implement stricter content reviews. These efforts can enhance the dissemination of accurate and comprehensive medical information, ultimately improving public health outcomes. Future research should continue to monitor content quality and explore additional strategies to ensure reliable health information reaches the public.

## Supplementary Information

Supplementary Material 1.

## Data Availability

The data sets generated during and/or analysed during this study are available from the corresponding author on reasonable request.
