# Supplementary material for: Assessing the content and quality of gastrointestinal endoscopy videos on TikTok, Bilibili, and Kwai: a cross - sectional study
Source: BMC Gastroenterol. 2026 Jul 13;26:615. doi: 10.1186/s12876-026-05108-6 (PMC13640380; doi:10.1186/s12876-026-05108-6)
Supplement: Supplementary file 1 [file 12876_2026_5108_MOESM1_ESM.docx]

Supplementary table 1. Global Quality Score (GQS) benchmark criteria.

| Score | Global Score Description |
| --- | --- |
| 1 score | poor quality, poor traffic, most information missing, not of any use to the patient |
| 2 score | Generally poor quality with poor flow, some information listed but many important topics lacked, of very limited use to patients |
| 3 score | medium quality with suboptimal flow, some of the main information was fully discussed but other information discussed insufficiently, somewhat helpful to patients. |
| 4 score | Good quality and generally good flow, most of the relevant information is listed, but some topics not covered, useful to patients. |
| 5 score | Excellent quality and excellent flow, very useful for patients |

Supplementary table 2. The Journal of the American Medical Association (JAMA) benchmark criteria.

| Score* | Score component | |
| --- | --- | --- |
| 1 score | Authorship | Provided authorship information |
| 1 score | Attribution | Listed copyright information and references/sources |
| 1 score | Currency | Included the initial date and subsequent updates |
| 1 score | Disclosure | Disclosed any potential conflicts of interest, funding, sponsorship, advertising support or video ownership |

*The criteria of each aspect were scored separately, and 1 point was accumulated when the criteria were reached. A total reliability score ranging from 0 to 4 was obtained.

Supplementary table 3. Modified DISCERN benchmark criteria.

| Score* | Reliability Score |
| --- | --- |
| 1 score | The video was clear, concise and understandable |
| 1 score | The information sources were reliable |
| 1 score | The information presented was balanced and unbiased |
| 1 score | Additional sources of information were provided for patient reference |
| 1 score | Areas of uncertainty or controversy were appropriately addressed. |

*The criteria of each aspect were scored separately, and 1 point was accumulated when the criteria were reached. A total reliability score ranging from 0 to 5 was obtained.
